# Is Traditional Agriculture a Viable Solution for Controlling Obesity and Food Insecurity in the Context of Climate Change? A Case Study from the Federated States of Micronesia

**DOI:** 10.5334/aogh.4761

**Published:** 2025-12-12

**Authors:** Sandeep Kandikuppa, Emihner Johnson

**Affiliations:** 1 Living Landscapes, India; 2 Island Food Community of Pohnpei, Federated States of Micronesia

**Keywords:** traditional agriculture, food security, institutional analysis, implementation research, Micronesia

## Abstract

*Background:* Climate change poses a significant threat to food security in the Federated States of Micronesia (FSM) by disrupting fisheries and global rice supply chains. Rice, an entirely imported staple, dominates local diets and is associated with a high prevalence of non‑communicable diseases (NCDs), particularly obesity and diabetes. Reviving traditional foods such as swamp taro has been proposed as a strategy to improve nutrition, reduce NCDs, and enhance climate resilience.

*Objectives:* This study examines whether traditional agriculture—specifically the promotion of swamp taro flour—can serve as a viable intervention to address obesity and food insecurity in FSM. It aims to identify the social, cultural, institutional, and environmental barriers and enablers influence the sustained uptake of traditional starches.

*Methods:* Using a case study approach focused on Pohnpei, the study applies the Consolidated Framework for Implementation Research. It combines analysis of secondary quantitative data on NCDs, key‑informant interviews, and a review of relevant national and state‑level policy documents to assess the implementation and outcomes of an intervention led by the Island Food Community of Pohnpei.

*Findings:* The uptake of swamp taro flour is shaped by complex interactions among historical preferences for rice, taste and convenience, supply‑chain constraints, gendered labor roles, limited institutional support, and weak community ownership of intervention infrastructure. Despite widespread availability and strong nutritional benefits, swamp taro remains underconsumed, while rice and processed foods continue to dominate diets.

*Conclusions:* Traditional agriculture can contribute to addressing obesity, food insecurity, and climate adaptation in FSM, but only if interventions account for the intertwined social, cultural, economic, and institutional factors shaping food choices. Institutional analysis, community participation, supportive public policy, and culturally sensitive communication are critical to scaling and sustaining such efforts in Pacific Island contexts.

## A Growing Crisis in Micronesia

Noncommunicable diseases (NCDs) like obesity and type‑2 diabetes are on the rise in the island nation of the Federated States of Micronesia (FSM). Studies indicate that more than eight‑tenths of the Micronesian population is either overweight or obese and that more than a quarter of the population reported having type‑2 diabetes [1]. The government and civil society organizations in FSM are seeking solutions to this explosion in NCDs. A solution that has gained traction is to encourage the population to replace rice and processed foods like ramen, which are the current staples consumed by the location population, with traditional foods such as swamp taro and breadfruit. Studies have shown that both these crops are rich in dietary fibers, resistant starches, vitamins, and minerals and are therefore likely to aid in weight loss [2, 3]. The theory is that increasing the consumption of traditional produce in Micronesia can improve diets and address the rising incidence of NCDs, including obesity. However, replacing rice and processed foods with traditional food crops such as taro or breadfruit has proven to be challenging. In this case study, *we delineate the barriers and enablers to the sustained uptake of traditional agricultural produce.* We ask: what are the factors that aid or hinder the consumption of traditional foods such as swamp taro, the stubborn persistence of rice, and the uptake of processed foods like ramen?

Although the government and civil society groups have been working to promote healthier options, such as swamp taro and breadfruit, and to make traditional crops easier to prepare, the consumption of rice remains high. Further, in recent decades, processed foods like ramen, made with refined flour and sugar, have also gained a lot of traction in FSM. In addition to being a public health concern, rice and processed foods threaten to undermine the climate change adaptation capabilities of the people of FSM. On the one hand, by disrupting the global rice supply chains, climate change has a deleterious effect on the ability of the people in FSM to access their staple food, rice. On the other hand, by making it harder to access rice, climate change is likely to push an ever greater number of people toward the other affordable source of calories in the island nation, viz., processed foods. Given the voluminous evidence about the direct correlation between processed foods and NCDs, we posit that climate change threatens to exacerbate the already significant NCD problem in FSM.

This looming crisis is well recognized within the policy circles of FSM. The National Adaptation Plan of FSM identified reducing the dependence on rice and processed foods as a key strategy to adapt to climate change [4]. Further, the Nutrition Action Plan of FSM recognizes that weaning the population away from white rice and processed foods is the key to improving the nutritional standards of the island nation. Thus, reviving the consumption of traditional foods is a key policy pillar from a public health and from a climate adaptation standpoint.

In our case study, we analyze an intervention by the Island Food Community of Pohnpei (IFCP), a nonprofit in FSM, to revive the consumption of traditional foods such as swamp taro, breadfruit, and local bananas. We especially focus on their efforts to revive the consumption of swamp taro (*Cyrtosperma merkusii*), a crop that is widely grown and easily available across FSM. A major innovation introduced by IFCP was to develop a flour made with giant swamp taro, which made it easier to use when compared to its natural form. Evidence suggests that when grains/starches are turned into flours, the odds of their consumption improve significantly [5, 6]. In taking a close look at this initiative, we tease out important lessons that can in turn help the government of FSM and civil society actors in designing better programs to push the people toward healthier diets. In our case study, we focus on one of the four main islands in FSM, Pohnpei, where IFCP has worked extensively to revive the consumption of taro and other traditional food commodities. A note about the methods: a major challenge in tracking health outcomes, including NCDs, in FSM is the paucity of long‑term and consistent data. To overcome this challenge, we combined data from multiple sources, including secondary data on NCDs from the STEPwise Approach to NCD risk factor surveillance (STEPS) [7] data from qualitative interviews with key informants, and perusal of key policy documents and reports published by the government of FSM and reports of IFCP.

Our case study is an important contribution to the literature on public health. First, it highlights how countries in the Pacific are struggling with NCDs, and how these struggles have been exacerbated by climate change. Second, it centers the experiences of the small island nations in the Pacific in the ongoing conversations about climate adaptation. In doing so, it highlights the unique challenges faced by many of these countries owing to their small economies, remoteness, and limited public health facilities and disease‑tracking capabilities of their governments. Third, it directly addresses two important public policy concerns of FSM and indeed of other island nations in the Pacific, namely, rising incidence of NCDs and climate change. Lastly, we use the Consolidated Framework for Implementation Research (CFIR) to analyze the connections among climate, NCDs, and traditional agriculture, and thereby examine how the interplay of social, cultural, economic, and ecological factors influences health and adaptation‑related outcomes.

### NCDs, climate change, and traditional agriculture: a survey of literature

Grecni, Bryson, and Chugen [8] predict that extreme rainfall and tropical cyclones in Pohnpei would cause flooding and landslides. These extreme events would damage standing crops and increase salinity on agricultural land, making farming difficult. It is further predicted that climate change will increase ocean acidification, damaging coral reefs, depressing fish availability [9, 10], and deepening food insecurity. Lastly, sea‑level rise is expected to contaminate freshwater resources and increase salinity on land [8]. But the biggest climate related threat to FSM emanates far from its shores. The heavy dependence on imported rice mostly from Southeast Asia, and on processed food sourced from the USA, Southeast Asia, Fiji, and Australia, renders FSM vulnerable to fluctuations in the global commodity prices [11]. Climate change is expected to have an especially detrimental impact on global rice production. Shifts in rainfall and temperature patterns are expected to depress rice production by about 4.2% globally, including in those countries from where FSM imports [12, 13]. The resultant fluctuations in the price of rice globally will likely disrupt the supply chains for countries like FSM and trigger food price inflation. The other staple food in FSM other than rice is processed foods mainly spam, other canned meat, refined flour, and ramen. The consumption of these foods is directly correlated with the incidence of NCDs worldwide. Climate change is expected to increase the dependence on processed foods in FSM. As global rice supply chains are disrupted and the price of rice increases, the people of FSM are likely to turn toward processed food like ramen in ever greater numbers. In this way, climate change is expected to contribute to a further upsurge in the incidence of NCDs. Over the past seven decades, the Micronesian diet has shifted from traditional foods such as taro and breadfruit toward imported and processed foods such as white rice, ramen, refined flour and processed meats like spam. This shift has addressed the problem of hunger but undermined the health of the population. FSM government statistics reveal a country grappling with high incidence of NCDs [14]. This trend is only likely to get accelerated in the face of climate change.

Micronesians consume a significant amount of rice, averaging 84 kg per year [15]. About 94% of the population eats rice at least thrice a week [16]. Further, in recent studies, a significant proportion of the population (about 67% in Pohnpei) reported that half or more of their diet was made up by imported foods including rice and processed foods [17]. Connell [16] shows that the regular consumption of rice and processed foods has resulted in a significant increase in the incidence of obesity, type‑2 diabetes, and other NCDs in FSM [18, 19]. Traditional crops such as the giant swamp taro and breadfruit that are locally grown and widely available [3, 20] in FSM, including Pohnpei, are known to be far more nutritious than rice. Studies show that swamp taro is rich in dietary fiber and resistant starches that aid in healthy digestion and weight management [21]. It is also rich in vitamins E and B6 and in minerals such as potassium and manganese, which improve metabolism [22]. These nutritional properties of swamp taro help in weight loss [23]. Swamp taro is a hardy crop that usually grows in rainfed regions and on the edges of swampy areas in FSM [19]. Swamp taro also has tremendous cultural significance for the people of FSM and is an integral part of events like death feasts [19]. Encouraging swamp taro consumption, as proposed by the government and IFCP, is a good strategy for addressing the growing obesity problem in FSM. Yet, despite its numerous, and well‑documented, nutritional benefits and widespread availability, consumption of swamp taro (and other traditional crops like breadfruit) remains low, if not on the decline. The question then is, why do the people of Pohnpei and FSM at large continue to consume rice and processed foods in lieu of swamp taro and other traditional foods? And how can the people of FSM be convinced to make the switch to traditional food crops?

To answer this question, we use the CFIR framework [24, 25] to (a) delve deeper into how institutional factors might be underpinning the NCD‑related outcomes observed in Pohnpei; (b) uncover the social, economic, institutional, and cultural factors underpinning sustained uptake and consumption of traditional foods; and (c) identify the motivations and concerns of people in Pohnpei with regard to the the uptake and use of traditional starch sources like the giant swamp taro.

### Study location

The FSM, an island nation comprising over 600 islands, with a total landmass of 700 sq. km is spread over 3.2 million sq. km of Pacific Ocean. Pohnpei is the largest island in FSM, the home to its capital, and has a population of nearly 40,000 people. The island of Pohnpei, and FSM at large, reports high incidence of NCDs and is also vulnerable to climate change.

### Data and methods

There is a shortage of systematic data on NCDs from FSM. The World Health Organization administered the STEPS, a “simple, standardized method for collecting, analyzing, and disseminating data on key NCD risk factors in countries” [7]. But the microdata is only available for the public for the years 2002 and 2008 [26, 27]. By 2019, this survey had been folded into the Hybrid Survey, but the microdata was not made available to the public when this article was authored. For this paper, we took the household‑level data for the available years, 2002 and 2008. We draw the figures for the 2019 Hybrid Survey from the report published by the Department of Health, government of FSM. The STEPS survey gathers measures such as BMI, blood‑pressure readings, fasting blood‑glucose level, waist size, and cholesterol levels [27]. Using this data lets us learn about the socioeconomic characteristics of the people who reported having NCDs in Pohnpei. We can also gain insights into the diet*ary* habits of the various population segments on the island, especially those who consume traditional starches like the giant swamp taro regularly.

The survey however has a major shortcoming; it does not capture the mechanisms that underpin the food choices of the Micronesians and as to why they prefer rice to traditional foods. To understand these mechanisms, we undertook key‑informant interviews (KIIs) in 2023. The informants were selected purposively based on consultations with IFCP and based on a determination by the authors, as to those who are best positioned to inform us about the questions tackled in this paper. The protocol and schedule for the KIIs were given human‑subject clearance by the Internal Review Board of the East–West Center, Honolulu.

We conducted the interviews as part of a baseline building exercise by the IFCP, a local nonprofit, to understand the factors that underpinned the strong preference for rice demonstrated by the people of Pohnpei. In all, we conducted 10 in‑depth semistructured interviews with the key field staff of IFCP (2), functionaries of the Department of Health, government of FSM (4), and some community leaders (4). All interviewees provided with informed consent that has been duly filed by the lead investigator in a password‑protected cloud file for future reference. The interviews were recorded, transcribed, and anonymized by the lead investigator before their content was analyzed using Atlas.ti software.

The theory of change that we test in this paper is that *the easy availability of and access to swamp taro and breadfruit encourages the people of Pohnpei to switch their food preferences from rice and processed crops to these traditional crops*. The KIIs helped us in understanding the material pulls and pushes that kept the people of Pohnpei locked into consuming rice and processed foods.

Lastly, we supplemented the survey data and KIIs with an analysis of important policy documents published by the government of FSM and IFCP. These documents were purposively selected in close consultation with the field partner, IFCP, and after discussions with personnel from the Department of Health and Social Services, Pohnpei State Government. The choice of the documents was also determined by their availability in the public domain. The policy documents thus chosen include the Pohnpei High School Survey Report [28], the Pohnpei Adult Hybrid Survey Report [17], FSM Multisectoral Plan of Action for Nutrition (MPAN) [29], and FSM Guidelines for Healthy Living [30].

To understand the conditions under which the Pohnpei’s people might switch from rice to swamp taro, we used the CFIR. This framework helps us in examining the complex factors including institutional structures, social norms, policies, programs, and cultural practices that underpin the food choices of the people of Pohnpei.

### NCDs in Pohnpei: a statistical overview

**Figure 1:**
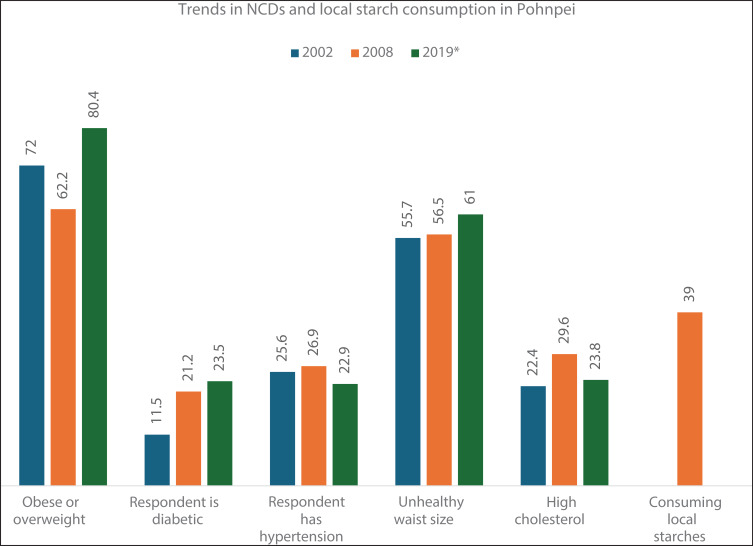
Trends in NCDs and local starch consumption in Pohnpei.

In this section, we use the microdata from the STEPS for the years 2002 and 2008, and the more recent aggregated data from the Adult Hybrid Survey report for the year 2019, to discuss the incidence of NCDs in Pohnpei. In doing so, we answer two questions: (a) what are the characteristics of the people who report NCDs? (b) And what is the current extent of traditional starch consumption on the island? STEPS data (see Figure 1) shows that 55% of women and 44% of men in FSM are obese (BMI ≥ 35). In Pohnpei alone, nearly 78% of the total population is either obese or overweight (BMI ≥ 25) with the percentage being as high as 82% among women (DoH, 2019). Compared to the BMI in 2002, this is an eight‑percentage point increase.

**Table:** 

	**Definitions**
Obese or overweight	% of respondents has BMI ≥ 25
Respondent is diabetic	% of respondents on meds or ≥ 126 mg/dl
Respondent has hypertension	% of respondents on meds or ≥ 140/90
Unhealthy waist size	% of respondents with waist is > 35 for women and > 40 for men
High cholesterol	≥ 190 mg/dl
Consuming local starches	% of HHs eating local starches at least thrice/week

The data also indicates that in Pohnpei about 61% of the population has an unhealthy waist size, defined as above 40 inches for men and above 35 inches for women. The percentage of women with an unhealthy waist size, at 84%, is significantly higher than that of men. FSM as a whole, and Pohnpei in particular, also reports significant incidence of diabetes (~23%). A quarter of the adult population below the age of 55 years reported to be diabetic (~25%). About 23% of the adult population of Pohnpei reported having hypertension, which is twice the percentage in 2002. The incidence of hypertension, having an unhealthy waist size, and having high cholesterol also remain high at 22.9%, 61%, and 23.8%, respectively, in 2019. STEPS microdata from 2002 and 2008 shows that women in Pohnpei were significantly more likely to be overweight or obese, with odds 115‑percentage points higher than men. Additionally, they had an 18‑percentage point higher chance of having an unhealthy waist circumference.

We find that 34% of the Pohnpeians never consumed traditional starches and about 28% did so 1–3 times a week. Only about 39% of the population in Pohnpei consumed traditional starches for four days or more in a week in 2008. STEPS data for 2008 further indicates that women and younger people were significantly less likely to consume traditional starches, relative to men and older people, respectively. Further, data from Adult Hybrid Survey 2019 indicates that nearly 68% of the population in Pohnpei consumes imported foods—mostly white rice and processed foods such as ramen, refined flour, and sugar‑laden foods—in half or more of their meals. This survey further indicates that the consumption of rice and processed foods is almost even across both genders and that the younger and those with higher education are more likely to consume these foods relative to older people and those with less formal education, respectively [17]. It is hard to ascertain temporal changes of the consumption of traditional starches due to a paucity of data. But it is reasonable to say that despite the best of intentions of the government and the nonprofits, rice and processed foods like ramen continue to dominate the palates of the people of Pohnpei, given that these are consumed at least once a day by almost everyone on the island [28].

It is clear from the STEPS data that the incidence of NCDs, especially that of obesity, is high on the island of Pohnpei. It is also evident that the percentage of households consuming traditional starches like giant swamp taro is low. Available evidence suggests that these traditional crops can help the Micronesians fight the growing incidence of NCDs. Furthermore, these crops are easily available across Pohnpei, having access to a taro patch and to a breadfruit or banana tree. Then, why is it that the consumption of these crops is lagging that of rice?

### Reviving swamp taro: a promising intervention

Climate change threatens the long‑term food security of FSM, including Pohnpei. Global warming is likely to increase ocean acidification that in turn is expected to accelerate coral bleaching. For FSM, which depends on the rich marine biodiversity in its coral reefs for its food security, ocean acidification poses a serious threat. Climate change also poses a significant threat to rice production in key rice‑producing countries such as Singapore, Japan, and the United States [11]. These countries are major suppliers to the FSM, and production shortages in these countries would make rice more expensive for import‑dependent countries like FSM, in turn resulting in food price inflation on the island. Given the already high preference for processed foods such as ramen and refined flour among the people of Micronesia, the contraction in the availability of rice is likely to drive more people toward these unhealthy options. Thus, there is a good chance that the uptake of processed foods will be even higher in the years to come, particularly in the face of climate change and all its attendant risks. FSM imports most of its staple foods including rice, refined flour, canned meat, ramen, and other processed foods, and a global disruption of rice production is likely to prompt the country to increase its reliance on these foods. Considering that there is already a significant uptake of these foods among the people of FSM, such a switch is unlikely to meet much resistance. Thus, it is likely that climate change might push the population of FSM toward consuming imported processed foods in ever greater quantities.

To address the long‑term threat to the food security of FSM that climate change posed and to wean away people from their dependence on imported rice and processed foods, the IFCP, in 2008, commenced efforts to revive the consumption of giant swamp taro and other traditional crops such as breadfruit and bananas. The tall broad‑leafed swamp taro plants are ubiquitous across Pohnpei, growing close to streams and other water bodies (see Picture 1 and Picture 2 for the plant and the corm, respectively).

**Picture 1:**
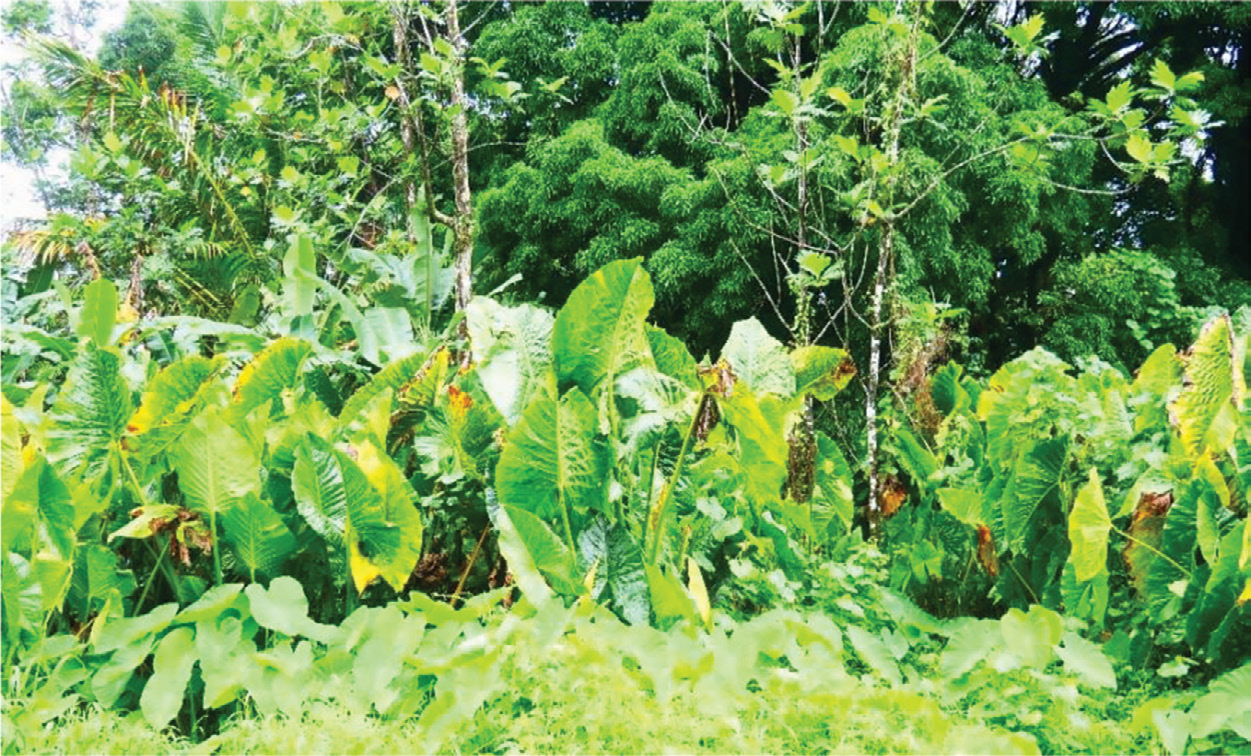
Giant taro plant with its characteristic broad leaves (photo credit Sandeep Kandikuppa).

**Picture 2:**
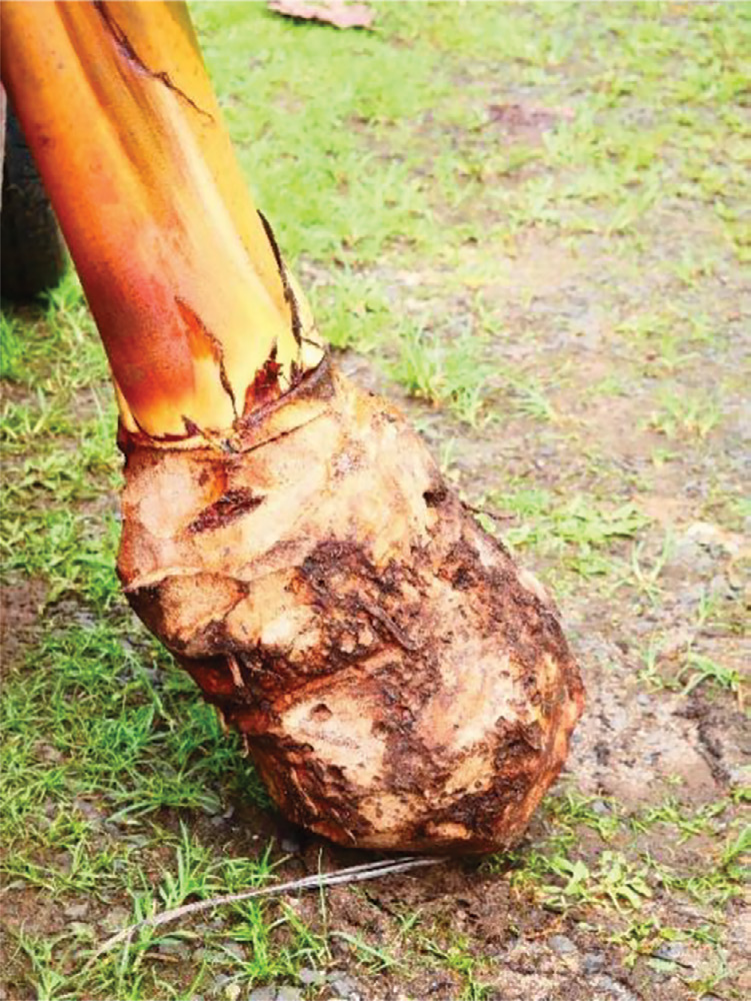
Corm of giant taro (photo credit Sandeep Kandikuppa).

IFCP tried to popularize the consumption of giant swamp taro by converting the natural corm into flour, generating awareness about the health benefits of taro, and by highlighting the cultural significance and the economic advantages of consuming this food regularly. Swamp taro flour is made by shredding and sun drying the corm and then running it through a flouring mill. As IFCP staff said during in‑depth interviews, “people would often say it takes only 30–45 minutes to cook rice. Taro takes forever.” But by grinding taro into flour, IFCP believed that it would become as easy to cook as rice, thereby making it accessible for a wider cross section of the population. Further, given that the people of Pohnpei were accustomed to consuming all‑purpose flour, it was felt that they would be more receptive to trying taro flour.

Toward this end, starting 2016, IFCP installed 16 flour‑milling machines in various municipalities on Pohnpei (please see Picture 3). A few more were installed on the island of Chuuk. In all, 22 machines were installed across various islands of FSM in a phased manner. While the initial set of machines were either manual or gas‑powered, the ones installed subsequently ran on electricity. Each machine could process up to 200 pounds of swamp taro in a day. In each case, the machine was installed in either the municipal office building or in another publicly accessible location.

**Picture 3:**
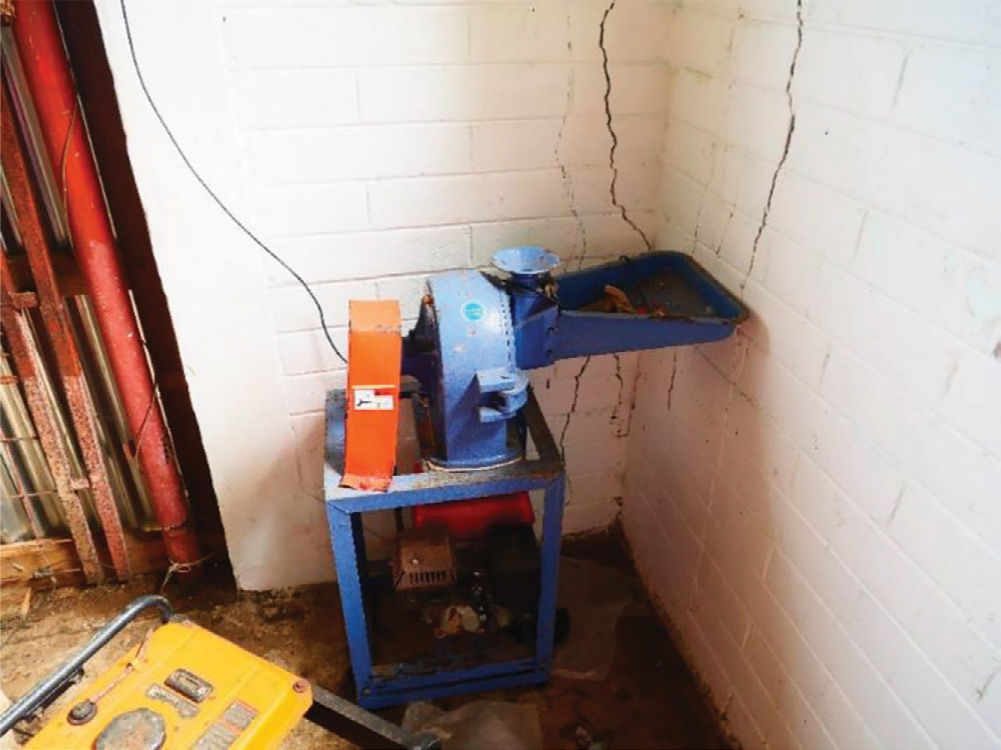
A gas‑powered flour mill in Madolenihmw municipal office (photo credit Sandeep Kandikuppa).

In a few cases, the flour mills were installed in private homes, but with the understanding that those wishing to access could do so with reasonable conditions. Alongside installing the machines, IFCP informed the users about the dos and don’ts of using the mills. The mills could only be used for dry goods which meant that swamp taro had to be sun‑dried to eliminate all moisture before being milled. Even a small amount of moisture could cause the machine to break down. Another critical part of this intervention was to highlight the health benefits of swamp taro and other traditional crops. IFCP made efforts to scientifically document the nutritional value of the traditional crops. The results of the studies established that the traditional crops had significantly higher quantities of nutrients such as fiber, vitamins, and other micronutrients that improve metabolism and contribute to weight loss. IFCP prepared dissemination material such as posters and flyers that were displayed prominently in the office and at events that IFCP organized.

The third crucial aspect of the intervention by IFCP was to celebrate Micronesian cuisine on every occasion. For instance, IFCP would volunteer to provide catering for events hosted by FSM government, where they would serve traditional cuisine including included swamp taro, breadfruit, and local bananas instead of rice. In doing so, IFCP hoped to reignite pride for local food in Pohnpeians. Closely related were efforts to unearth, document, and repopularize traditional cuisines of Micronesia, especially dishes made out of taro and other traditional starches. Lastly, IFCP strove to develop recipes using taro flour. For instance, they developed taro bread that they supplied to some of the grocery stores and restaurants in Pohnpei. They sought to demonstrate that taro flour was easy to use and that it could be adapted to suit modern‑day palates.

In the next section, we discuss the factors that aided or hindered the uptake and the sustained consumption of giant swamp taro on the island of Pohnpei.

### “Taro is for pigs”: why Pohnpeians do not eat their taro

**Figure 2:**
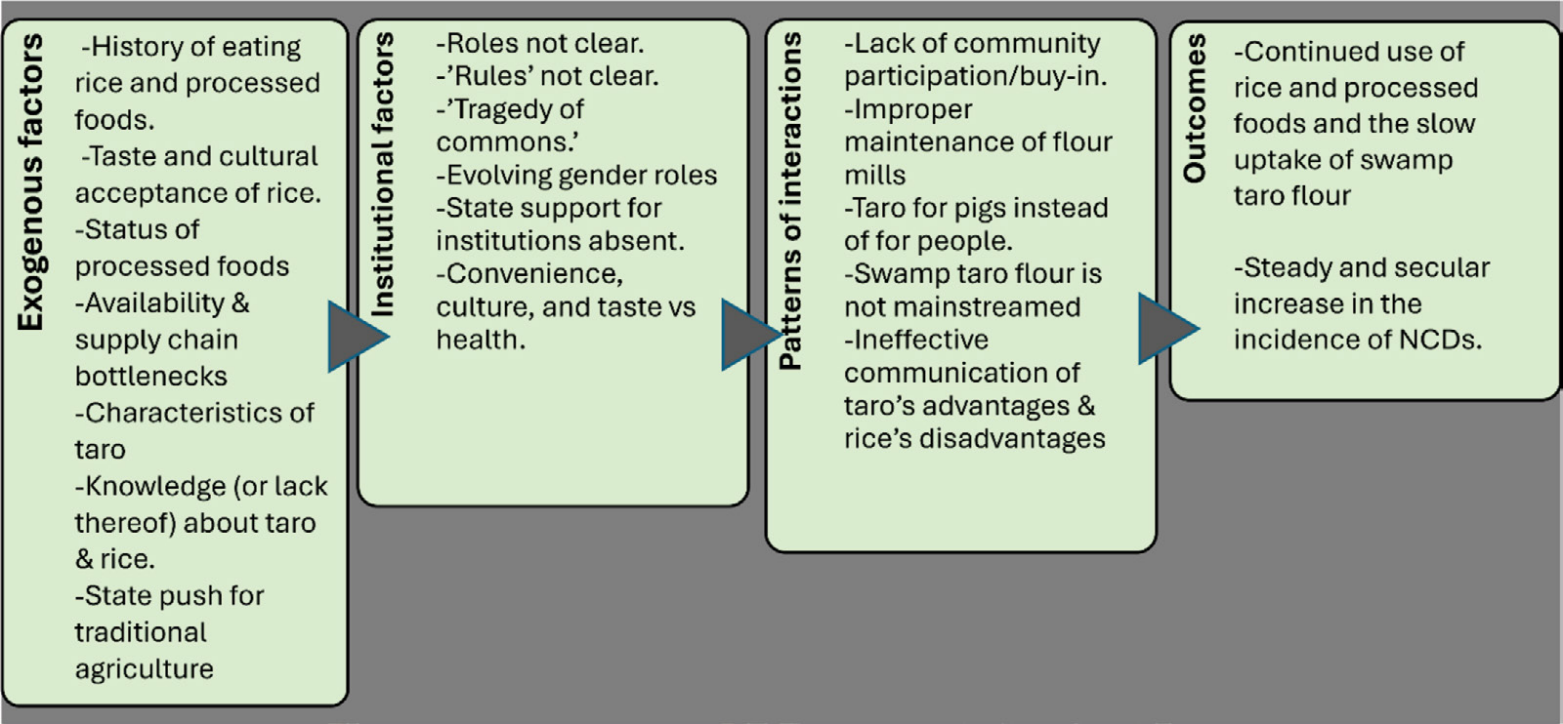
Factors underpinning consumption patterns of swamp taro.

Notwithstanding its numerous health, environmental, and economic benefits, and despite the efforts of IFCP to make it more accessible, swamp taro has not gained much traction with the population of Pohnpei. Understanding what is holding back the sustained uptake and consumption of taro and other traditional foods by the people of FSM is valuable, given their importance from climate adaptation and a public health standpoint. We use the CFIR to understand the multiple factors that shaped the outcomes of IFCP’s interventions (see Figure 2). In the figure, the exogenous factors that interact with the institutional factors engender unique patterns of interaction that then result in specific outcomes, namely, the continued consumption of rice and processed foods and the resultant inexorable increase in NCDs [31].

#### Exogenous factors

First among the exogenous factors influencing the outcome of IFCP’s intervention is the long history of rice consumption in FSM. The island nation was first introduced to rice in the 1940s when it came under the Japanese occupation during the Second World War. Following the end of the War, when the governance of Micronesia passed on to the USA, the former saw continued influx of surplus rice produced in the latter. More than 80 years of conditioning have changed the food habits of the Micronesians to a point where today a meal without rice is almost unthinkable. The next major shift in the food habits of FSM came in mid‑ to late‑twentieth century when there was an uptick in the import of processed foods mostly from USA, Fiji, and Southeast Asia, high in salt, fat, and sugar increased manifold. Extant literature on the subject surfaces the key drivers of this trend. First, the migration of a large number of people from FSM to the USA for jobs, education, or health care precipitated labor shortages within the country which in turn made it difficult for the locals to harvest traditional crops such as giant swamp taro and breadfruit [11]. Further, in the late‑twentieth century, FSM and other countries across the Pacific pursued trade liberalization that increased the inflow of processed foods mainly from Southeast Asia, Fiji, and the USA. Rice and ramen (and indeed other processed foods) emerged as convenient alternatives to traditional foods such as swamp taro and breadfruit, which were relatively more difficult to harvest and prepare for consumption. Conversely, the ease of accessing rice and processed foods made swamp taro and other traditional foods inconvenient choices. And to the detriment of the efforts of the Island Food Security of Pohnpei, this lack of preference for swamp taro seemingly extended to taro flour as well.

This brings us to the next set of exogenous factors that shaped the outcomes of IFCP’s interventions, namely supply. Even though rice and processed foods are imported by FSM, they are available in almost every neighborhood department store in the island nation. In every restaurant in Pohnpei, including those serving traditional Micronesian dishes, rice and processed foods such as spam and ramen are staples on their menus. Most of the schools and educational institutions also serve rice as part of their subsidized student‑lunch programs. The widespread availability of rice and processed foods makes it easier for people to consume them. The availability of these foods is connected to and also driven by the next exogenous factor, peoples’ preference for ramen and rice. As the interlocutors for this study admitted, the tastes and preferences of the people of Pohnpei and FSM, young and old, have shifted firmly in the direction of rice and processed foods. Except those above the age of 60 years, an overwhelming majority of the rest of the population prefers to consume white rice on a daily basis. Processed foods enjoy an aspirational status in the Micronesian society. A less understood aspect of this particular trend is how the USA itself is perceived by the Micronesian society. For most of the young people, living in the USA is aspirational. The Compact of Free Association between FSM and the USA makes for unfettered and visa‑free flow of people between these two countries. As a result, more and more people aspire to live in the USA and “make it,” as one of the IFCP staff put it to me. As a result of underlying social current, people seem “American” with progress. As one of the community leaders put it to me about what young people should do, “they should go the USA and make a living. If needed, they should join the Army [American] and experience the world. That would make them [the Micronesian young men] men.” Processed foods are seen as symbols of American advancement, even though most of these foods today come from Southeast Asia. Young people, including school‑going children, consider ramen to be both a delicacy and a “cool” dish. As an official from the Department of Health and Social Services said,

“young kids today like to eat ramen or noodles. If someone can really pay, then they go and have expensive sushi. Even if their parents pack them coconut boiled taro, they still prefer to buy ramen from the school canteens. So, the schools also cater to their demands.”

Conversely, swamp taro is seen as old fashioned. Another factor that drives people away from swamp taro is the labor‑intensive harvesting process that is involved. The heavy corms must be harvested from a field that may not always be located close to one’s home. Further, one needs the helping hands of friends, family, and neighbors to harvest the swamp taro in sufficient quantities. However, the growing out‑migration of the Micronesian population, mostly to the USA, makes it difficult to find people to help with the harvesting. This makes it difficult for people to consume swamp taro except on special occasions. On the other hand, the convenient availability of rice, conversely, and lack thereof of swamp taro, is one of the factors why people continue to prefer the former. The supply‑related difficulties corresponding to swamp taro also extend to swamp taro flour.

There are two intertwined problems with respect to the availability of swamp taro flour. First, while the process for making swamp taro flour is well established, it is not clear as to who will produce it, how it will be supplied to the points of sale, and how much money will flow into the pockets of those who produce it. Instead, those who want taro flour must procure their own swamp taro and find a mill to grind it into a flour. Second, and related, is the lack of adequate flour mills on the island. There are 16 mills on an island of 40,000, mostly in central locations like a municipal office. For a consumer, it is easier, and cheaper, to buy rice from a neighborhood store than to locate a mill to grind their flour. This brings us to the next exogenous factor that influences the consumers’ choice between rice and taro, the very characteristic of the crop. The giant corms, sometimes weighing as heavy as 25 kg each, would have to be cut into manageable pieces and then cooked for a long time. This in turn requires considerable amount of firewood or butane, the two main fuels on the island. Collecting firewood is time‑consuming and energy‑intensive, and butane is expensive because it is entirely imported.

IFCP developed taro flour to circumvent the difficult cooking process. However, producing the taro flour itself is challenging. The corm has to be cut into smaller pieces, shredded, and sun‑dried before it is ready to be put into the mill. Moreover, while swamp taro can remain underground without getting spoilt for as long as 15 years, once harvested, the corm needs to be used quickly, within a matter of days. Or else, it starts rotting. Consequently, the corm has to be dried and milled as soon as it is harvested. In this scenario, rather than reducing it, taro flour could inadvertently be adding to the burden of the people responsible for making food within a household, who culturally have been women. The challenge is compounded by the fact that people have to harvest their own taro and take it to the mill. This combined with the reality that only a handful of places on the island sell ready‑made taro flour, it is easy for the people on the island to access it. This complex of factors is a significant barrier to the sustained consumption of swamp taro flour.

The next exogenous factor underpinning the sustained use of swamp taro is the knowledge of using it. Swamp taro contains calcium oxalate [17], a bitter‑tasting compound, which can result in itchy mouth and throat if not cooked properly. From our interviews, it was clear to us that the recipes to cook taro properly were unknown to many people, especially the younger generations. Further, cooking swamp taro is a long and time‑taking process. Both these factors are likely deterring more people from adopting swamp taro in their diets on a regular basis. As one of the women we spoke to told us, “Cooking rice is faster. You can throw rice into it [electric rice cooker] and forget about it, come back after a white and eat it. Swamp taro can take forever. It requires a lot of time. I don’t have that kind of time.”

In 2008, the then President of FSM Manny Mori exhorted his fellow citizens [32] to eat more local foods instead of rice. Since then, traditional foods have been mentioned in two important policy documents of the island nation, the National Adaptation Plan and the Nutrition Action Plan, both published in recent memory. Yet, as one of the IFCP employees shared, whenever there is an event hosted by the government, rice is conspicuously served. And although taro and breadfruit are also served at such events, thanks to the efforts of civil society organizations like IFCP, most of the participants just gravitate toward rice. Further, despite being mentioned in key policy documents, there is no push programmatically, to educate the public about the benefits of consuming traditional foods. This lack of policy support in real terms could be another factor underpinning the lukewarm reception of traditional foods like swamp taro following IFCP’s intervention.

#### Institutional factors

All the aforementioned exogenous factors influence and feed into the institutional factors underpinning taro consumption. Reviving the consumption of traditional crops like swamp taro faces several institutional challenges. These include public apathy, the preference for convenience and taste over health, and the limitations of the supply chain. These factors have in turn fueled an indifference among the people of Pohnpei toward traditional foods like swamp taro, which might have extended to the interventions being undertaken by IFCP. Further, neither IFCP nor the government of FSM have envisioned ways to rope in community participation, whether it be in the management of the taro flour mills or in the promotion of traditional foods. For instance, when the flour mills were installed by IFCP, nowhere it was specified that who would be responsible for their maintenance and upkeep. Who would monitor their use? Who would step in if the mills suffered malfunction? And who would be responsible for imposing sanctions or fines if the mills were misused? Churches, citizens’ groups, local leaders, and community elders, to name a few, did not have a defined role in the project developed by IFCP. This was an institutional shortcoming.

Closely related, the “rules of the game” regarding the use of the flour mills were not clearly specified. The current process is that anyone who needs to use the mill goes to the municipal office and takes it to their homes. No clear responsibility has been assigned to anyone to oversee their use. What is the jurisdiction of each flour mill, that is, what is the area that each mill will cater to? Who can access the machines? How will they access them and how often? How would the inevitable conflicts about the use of the mills be resolved? These important questions were largely left unaddressed. This in turn has given rise to the “tragedy of the commons” problem. The flour mills that are a public good kept in widely accessible spots throughout Pohnpei often got misused and no one was assigned to maintain them. Some people used it to grind *sakau* root to make an intoxicant. Since the root has moisture in it, the flour mills were damaged, as they were designed only to handle dry goods. The failure to find answers to these questions seems to have hamstrung IFCP in its pursuit of reviving swamp taro.

The prevailing cultural norms, especially the evolving gender roles, are another crucial aspect that shaped the outcomes of IFCP’s intervention. In FSM, women are traditionally expected to cook. In many households, women are also responsible for gathering swamp taro from the taro patches. Swamp taro requires considerable effort at every stage of the preparation, from collection to chopping to ultimately cooking, and adds significantly to the workload of the women. Swamp taro flour can potentially reduce the workload of women as it is ready to cook. However, due to the supply‑chain challenges discussed above, taro flour is also likely to add to the women’s workload. In a country where more and more women are entering the labor markets, it is likely difficult for them to dedicate the time and effort needed to get swamp taro ready to eat. On the other hand, rice is easy to cook. This makes it a compelling alternative to swamp taro and to its flour.

Another important institutional factor that we identified is that the state has not extended any recognition to community‑level efforts, to revive traditional foods like swamp taro. Institutional scholars have argued that some kind of formal state recognition for the communities’ efforts at collective action is critical for the success of those efforts [33]. In FSM, even though the government has prioritized reviving traditional food crops, it has not taken explicit programmatic actions to further their revival. Nor it has extended any formal recognition to the efforts being put in community‑based organizations like IFCP. Consequently, these efforts could not be scaled up.

#### Patterns of interaction

The absence of clarity over the role played by the stakeholders has resulted in the intervention not getting much buy‑in from the community at large. This lack of community buy‑in can also be attributed to the other social, economic, and cultural factors discussed above. These factors together have given rise to specific patterns of interaction which have in turn shaped the outcomes of the swamp taro intervention. A key pattern of interaction that has likely shaped the outcomes of IFCP’s intervention is how people perceive swamp taro. As we were interacting with Stephen (name changed), a respected traditional leader hailing from one of the wealthiest families on the island, he alluded to the cultural significance of the crop. He pointed out that swamp taro is integral to the gifts that are given to traditional leaders on the island during important local festivals. However, in their day‑to‑day lives, a number of people feed taro to their pigs. There are two reasons for this. First, taro is perceived as being primitive and as a symbol of backwardness. Conversely, since it comes from “developed” countries such as the USA and Japan, rice is seen as a sign of progress and therefore considered to be superior to taro and other traditional foods. There is a cultural stigma attached to the consumption of taro. Second, pigs have high commercial value and can provide the households that are rearing pigs with significant financial returns. And since rice is available so easily and cheaply, it makes eminent sense for an average Pohnpeian household to feed the taro to their pigs and use the money thus earned to buy rice from the market. This amalgam of culture and economics is a powerful reason why swamp taro consumption has not picked up steam despite IFCP’s efforts.

We also found that communication by IFCP about the benefits of traditional crops has been ineffective and that these crops have not been mainstreamed in a significant way. Due to the unreliable supply chains of swamp taro, they seem to remain on the fringes of public imagination despite being almost ubiquitous on Pohnpei. Further, many people, particularly the younger generation, are unaware of the best ways to cook and consume swamp taro. As a result of these factors, IFCP has found bringing these crops into the mainstream challenging. And while IFCP has documented the benefits of swamp taro and other traditional foods extensively, it was hamstrung in effectively communicating these benefits to the wider public. Evidence indicates that if the messaging about the health impacts of food is too forceful or relies on negative imagery, especially when addressing issues like obesity, it can push people away from healthy eating options [34]. On the other hand, carefully crafted messages can push people to make healthier choices [35]. IFCP did its best to craft a coherent message with the limited resources that it had, about the health impacts of rice and, conversely, the health benefits of traditional crops like swamp taro. It published informative posters and flyers and published recipe books of dishes that use swamp taro and other traditional crops. Moving forward, these messages must be carefully evaluated for their effectiveness and strengthened as needed.

The exogenous and institutional factors, and the patterns of interactions discussed above, have contributed to the continued use of rice and processed foods, to the lukewarm response that swamp taro and other traditional crops receive, and to the unabated increase in NCDs like obesity.

### Ways forward and conclusion

Climate change increases the probability of the NCDs going up in FSM. As the shifts in climatic patterns threaten to undercut the fish production of FSM and jeopardize the global rice supply chains on which the food security of the nation utterly depends, this in turn increases the chances that the people of FSM will further increase their reliance on highly processed foods like ramen. This in turn threatens to increase the incidence of NCDs in the island nation that is already very high. Thus, both directly and indirectly, climate change threatens the long‑term food security of FSM. The efforts to revive the consumption of traditional crops such as swamp taro and breadfruit are therefore important to enhancing the capacities of FSM to adapt to climate change. If these strategies succeed, they can provide the people of FSM with a viable alternative to rice and processed foods that is local, sustainable, and nutritious. These traditional crops can, in addition to helping with climate adaptation, also help provide resolution to the NCD crisis that has already engulfed the island nation.

However, as this paper shows, there are inherent challenges to the uptake of these crops. The difficulties in procuring, processing, preparing, and consuming swamp taro is proving to be a powerful barrier to the widespread uptake of this crop as an effective alternative to rice or processed foods. In such a scenario, changing the long‑standing food habits of an entire country is challenging. However, as experience from countries like India shows, an approach that combines institution building, engagements with diverse stakeholders, and robust communication can achieve the desired results [36]. We used the CFIR framework to analyze IFCP’s intervention aimed at reviving traditional crops, such as swamp taro. Our analysis brought to light valuable lessons that can guide us in improving future interventions. First, we need to address the supply issues of taro and other traditional crops. It is important to ensure the availability of taro flour in as many stores as possible. Second, it is important to involve the community and ensure that the intervention is mindful of the cultural, economic, and social factors that influence the peoples’ food choices. This includes recognizing the unique needs of historically marginalized groups, such as women. Third, it is important to engage deeply with community leaders. In FSM, traditional leaders and community elders can provide critical knowledge input that could strengthen initiatives like IFCP’s and offer insights on how to communicate sensitively with various groups. Fourth, adopting an institutional approach that delineates clear boundary rules, and rules for appropriation, provisioning, monitoring, and sanctioning, would be critical. Fifth, it is important to seek recognition and support from the government for interventions like IFCP’s that aim to revive traditional foods. Lastly, such interventions must be supported by a robust information dissemination strategy attuned to the local population’s sensibilities. The road to climate adaptation and reducing the incidence of NCDs in FSM must pass through institutional building and collective action for the desired outcomes of reduced NCDs and robust adaptation to fructify. This is true not just for FSM but also for other island nations in the Pacific, many of which face similar challenges.

Using CFIR, our paper delineates the social, ecological, economic, cultural, and institutional factors that influenced the outcomes of IFCP’s interventions. In doing so, we reveal the need for systematic studies to test the efficacy of different institutional models to address the challenges that lie at the intersection of climate change and public health, in countries across the Pacific. In light of the existential threat posed by climate change to many of these countries, which comes on top of the other socioeconomic realities like low population and limited resources that they already confront, such studies can lead to new ways of imagining climate adaptation, rooted in traditional knowledge and communities’ ability to come together and take collective action.
